# Supplementary material for: Plasmodium falciparum parasites deploy RhopH2 into the host erythrocyte to obtain nutrients, grow and replicate
Source: eLife. 2017 Mar 2;6:e23217. doi: 10.7554/eLife.23217 (PMC5365316; doi:10.7554/eLife.23217)
Supplement: Supplementary file 2. — DOI: http://dx.doi.org/10.7554/eLife.23217.019 [file elife-23217-supp2.docx]

| **Oligo Name** | **Sequence (5'-3')** | **Target sequence** |
| --- | --- | --- |
| Generation and analysis of *P. falciparum* RhopH2-glmS line | | |
| DO227F | gcggccgcCATTCCGAATCTCACTGTCAAGA | RhopH2 CDS |
| DO228R | cg*ctgcag*gACTACTTTTTAAGATATACAAG | RhopH2 CDS |
| DO233R | ccgggacgtcgtacgggtatgc | HA epitope tag |
| DO276R | GTGATTTCTCTTTGTTCAAGGA | GlmS |
| DO354 | AGAGCAAACTTAAGATCCGT | RhopH2 CDS |
| Generation and analysis of *P. berghei* RhopH2 iKD line | | |
| DO62F | ccgatgctagcgtacgGCTTCCTCATGTTATTTCACATTT | RhopH2 5'UTR |
| DO63R | aatgccccggggcggccgcTTTAAAGATCATAAACAATAATTAA | RhopH2 5'UTR |
| DO291F | gcacctgcagATGGTAAAACTATCAGGGAT | RhopH2 CDS |
| DO67R | cgtgatatcgtacgctagcGAGCATTGTATAAGCATCCTTATC | RhopH2 CDS |
| DO173 | CGAGCCTGAATATATGACCTATGC | RhopH2 5'UTR |
| DO174 | GCGAAACGTAATAACATGATGC | RhopH2 CDS |
| Transcription analysis | | |
| DO420F | GCTGCATACATGCTAACAT | PbRhopH2 CDS |
| DO421R | AACATCTATTGCATCTTCA | PbRhopH2 CDS |
| MK22F | AGCAGCAACCACTGCCTTAT | PbEXP2 CDS |
| MK23R | CAAACCTGGATCGTGTCGTA | PbEXP2 CDS |
| DO605F | TTATGGATGCATGCAG | PbRhopH2 |
| DO616R | TTGAGTTACATTTCGGCA | PbRhopH2 |
| DO614F | ACAGTTTATGACACAGTTGGTC | PbRhopH2 |
| DO615R | GTCCATAATGTGTAATAGAAGTACT | PbRhopH2 |
| DO567F | AATTAAAGAAGCATCTGAGGGTCCAC | PbGAPDH |
| DO568R | TTGAATATCCCCATTCATTGTCATACC | PbGAPDH |
